# c-Cbl、Cbl-b和EGFR在非小细胞肺癌中的表达及其预后价值

**DOI:** 10.3779/j.issn.1009-3419.2011.06.17

**Published:** 2011-06-20

**Authors:** 昕 焦, 波 金, 秀娟 曲, 顺朝 闫, 科佐 侯, 云鹏 刘, 雪君 胡

**Affiliations:** 1 110001 沈阳，中国医科大学附属第一医院呼吸内科 Department of Respiratory Medicine, the First Hospital of China Medical University, Shenyang, 110001, China; 2 110001 沈阳，中国医科大学附属第一医院肿瘤内科 Department of Medical Oncology, the First Hospital of China Medical University, Shenyang, 110001, China

**Keywords:** 肺肿瘤, 表皮生长因子受体, c-Cbl, Cbl-b, 预后, Lung neoplasms, Epidermal growth factor receptor (EGFR), c-Cbl, Cbl-b, Prognosis

## Abstract

**背景与目的:**

表皮生长因子受体（epidermal growth factor receptor, EGFR）与肺癌的发展密切相关，其功能受泛素连接酶（Casitas B-lineage lymphoma, Cbl）家族调节，本研究旨在探讨c-Cbl、Cbl-b和EGFR在非小细胞肺癌（non-small cell lung cancer, NSCLC）组织中的表达及其在预后判断方面的应用价值。

**方法:**

采用组织微阵列联合免疫组织化学染色技术检测94例NSCLC组织中c-Cbl、Cbl-b、EGFR的表达，分析其与临床病理因素及预后之间的关系。

**结果:**

c-Cbl、Cbl-b和EGFR的阳性表达率分别为30.9%（29/94）、84.0%（79/94）和60.6%（57/94）。c-Cbl、Cbl-b蛋白表达与年龄、病理类型、TNM分期、淋巴结有无转移及吸烟史无关。EGFR、c-Cbl、Cbl-b的表达与患者的总生存无明显相关。亚组分析显示，在EGFR阳性组患者中，c-Cbl阳性组患者的总生存期（overall survival, OS）明显优于c-Cbl阴性组的患者（*P*=0.014）。

**结论:**

检测c-Cbl蛋白的表达水平可能有助于预测EGFR阳性NSCLC患者的预后。

## Abstract (en)

**Background and objective:**

Epidermal growth factor receptor (EGFR) is closely correlated with the progression of lung cancer. Its activity is modulated by Casitas B-lineage lymphoma (Cbl) family. The aim of this study is to investigate the expression and clinical relevance of c-Cbl, Cbl-b and EGFR in non-small cell lung cancer (NSCLC).

**Methods:**

Expressions of c-Cbl, Cbl-b and EGFR protein were detected with tissue microarrays and immunohistochemistry technique in 94 cases of NSCLC. The correlations between the expression of the three proteins and clinicopathological parameters were analyzed.

**Results:**

The positive expression rates of EGFR, c-Cbl and Cbl-b were 60.6% (57/94), 30.9% (29/94) and 84.0% (79/94), respectively. The expression of EGFR, c-Cbl and Cbl-b was not associated with age, pathological type, TNM stage, lymph node metastasis, and smoking history. c-Cbl and Cbl-b status was not significantly correlated with overall survival. Subgroup analyses showed that c-Cbl-positive patients had longer survival than c-Cbl-negative patients in EGFR-positive group (*P*=0.014).

**Conclusion:**

Detection of c-Cbl protein levels might contribute to the prognosis evaluation of EGFR-positive NSCLC.

表皮生长因子受体（epidermal growth factor receptor, EGFR）是受体酪氨酸激酶（tyrosine kinase, TK）erbB/ HER家族的一个成员，其在肺癌组织中高表达能够促进肿瘤血管生成、细胞增殖、粘附、侵袭及转移等^[1, 2]^。针对EGFR的靶向治疗研究是近年来肺癌治疗领域的一大研究热点。

泛素蛋白酶体途径是真核细胞内选择性降解蛋白质的重要途径，Cbl（casitas B-lineage lymphoma, Cbl）是一种泛素连接酶，在哺乳类中有c-Cbl、Cbl-b和Cbl-3三种，可通过特异性选择底物蛋白启动泛素化降解途径，参与受体和非受体酪氨酸激酶功能的负向调节^[3, 4]^。最新研究^[5]^发现在非小细胞肺癌（non-small cell lung cancer, NSCLC）组织中存在c-Cbl及Cbl-b的突变，参与NSCLC的发生与转移。在EGFR信号衰减的过程中，Cbl识别并泛素化EGFR，介导EGFR的泛素化降解^[6, 7]^。Shtiegman等^[8]^发现在NSCLC细胞中当EGFR特定位点发生突变而不能被Cbl降解时，会导致细胞增殖信号的持续活化，但尚无Cbl与EGFR相关性的临床报道。本研究旨在应用免疫组化方法检测NSCLC患者组织中EGFR、c-Cbl及Cbl-b的表达，并探讨表达水平与患者预后之间的关系。

## 1 材料与方法

### 1.1 标本来源

收集1997年2月-2000年9月在中国医科大学附属第一医院胸外科接受肺癌根治术并经术后病理证实为NSCLC且有完整随访资料的94例患者的石蜡标本。其中男性73例，女性21例；年龄34岁-73岁，中位年龄54.0岁。按世界卫生组织（WHO）1999年肺癌分类标准进行肺癌的组织学分类。TNM分期根据1997年国际抗癌联盟（UICC）标准。所有患者术前均未经任何抗癌治疗，随访截止时间为2005年9月。

### 1.2 组织芯片制作

将NSCLC标本制作成组织芯片（技术由上海芯超生物科技有限公司提供）。简要制作步骤：存档蜡块经病理专家复诊及组织定位后，应用组织阵列仪打孔取材（直径1.0 mm），每块取两点，制作成阵列块；应用切片机连续切片，厚度为4 μm；捞片，烤片后再次复诊，制作成可供免疫组化染色使用的组织芯片。

### 1.3 试剂和方法

鼠抗人c-Cbl单克隆抗体购自BD公司；鼠抗人Cbl-b及兔抗人EGFR单克隆抗体购自Santa Cruz公司，SP检测试剂盒及DAB显色液购自福建迈新生物技术开发有限公司。采用免疫组化SP法，按照SP检测试剂盒说明书所示步骤进行操作。PBS代替一抗作为阴性对照。

### 1.4 免疫组化结果判定

EGFR阳性染色为位于细胞膜/胞浆的棕黄色颗粒，c-Cbl、Cbl-b的判定以胞浆中出现棕黄色颗粒为阳性染色。显微镜400倍视野下随机选取5个有代表性的不同区域，每个视野的得分由阳性细胞的比例和显色强度两方面决定，依照阳性细胞比例 < 5%、5%-25%、25%-50%、50%-75%、>75%分别为0分、1分、2分、3分、4分；依照显色强度未着色、浅黄色、棕黄色、棕褐色分别为0分、1分、2分、3分；将两项得分相乘得到该视野的最终得分，选取的5个视野得分的平均值为最后得分。最后该切片按照得分分为以下4个等级：0分-1分为（-），2分-4分为（+），5分-7分为（++），≥ 8分为（+++）。（-）视为阴性，（+）-（+++）视为阳性；（-）-（+）视为低表达，（++）-（+++）视为高表达。

### 1.5 统计学方法

所有资料均用SPSS 13.0统计分析软件，组间差异采用χ^2^检验和*Fisher*精确概率法，相关性分析采用*Spearman*等级相关，生存数据采用*Kaplan-Meier*生存曲线分析及*Log-Rank*检验，并采用*COX*比例风险模型进行多因素生存分析。*P* < 0.05为差异有统计学意义。

## 2 结果

### 2.1 NSCLC组织中EGFR、c-Cbl、Cbl-b的表达

EGFR阳性染色为位于细胞膜或胞浆中的棕黄色颗粒（图 1A），c-Cbl、Cbl-b阳性染色为位于细胞浆的棕黄色颗粒（图 1B，图 1C）。EGFR、c-Cbl、Cbl-b三种蛋白在94例肺癌组织中的阳性表达率分别为60.6%（57/94），30.9%（29/94）和84.0%（79/94）。EGFR与c-Cbl、Cbl-b的表达无明显关系（*P*=0.931, *P*=0.866），c-Cbl和Cbl-b之间呈正相关（*r*=0.435, *P* < 0.001）。

**1:**
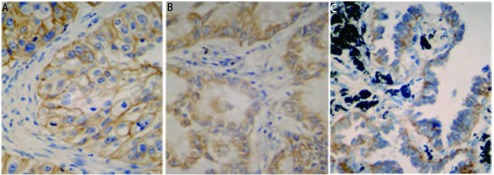
EGFR、c-Cbl、Cbl-b在NSCLC中的阳性表达（SP，×400）。A：EGFR；B：c-Cbl；C：Cbl-b。 EGFR, c-Cbl and Cbl-b positive staining in NSCLC (SP, ×400). A: EGFR; B: c-Cbl; C: Cbl-b.

### 2.2 NSCLC组织中EGFR、c-Cbl、Cbl-b的表达与临床病理特征的关系

女性患者EGFR阳性率为81.0%（17/21），男性为54.8%（40/73），女性患者EGFR阳性率高于男性（*P*=0.042）。EGFR、Cbl-b蛋白表达与年龄、病理类型、TNM分期及淋巴结有无转移、吸烟史等参数无关（表 1）。c-Cbl蛋白表达与肿瘤大小（T分期）存在负相关趋势，但无统计学差异（*r*=-0.192, *P*=0.078），与其它参数无关。

**1:** NSCLC中EGFR、c-Cbl及Cbl-b的表达与临床病理特征的关系 Relationship between EGFR, c-Cbl and Cbl-b expression and clinical pathophysicological characteristic of NSCLC

Parameter	*n*	EGFR		*P*	c-Cbl		*P*	Cbl-b		*P*
		Positive	Rate		Positive	Rate		High expression (%)	Rate	
Age (year)				0.593			0.056			0.770
< 60	59	37	62.7%		22	37.3%		22	37.3%	
≥60	35	20	57.1%		7	20.0%		12	34.3%	
Sex				0.042			0.876			0.215
Male	73	40	54.8%		22	30.1%		24	32.9%	
Female	21	17	81.0%		7	33.3%		10	47.6%	
Histology				0.969			0.849			0.518
Squamous cell carcinoma	41	25	61.0%		12	29.2%		12	29.3%	
Adenocarcinoma	47	28	59.6%		16	34.0%		20	42.6%	
Large cell carcinoma	5	3	60.0%		1	20.0%		2	40.0%	
Adenosquamous cell	1	1	100.0%		0	0		0	0	
carcinomas										
Differentiated				0.483			0.189			0.562
Highly&modrately	60	38	63.3%		22	36.6%		23	38.3%	
Poorly	34	19	55.9%		7	20.6%		11	32.4%	
Tumor size				0.241			0.078			0.408
T1+T2	70	40	57.1%		25	35.7%		27	38.6%	
T3+T4	24	17	70.8%		4	16.7%		7	29.2%	
Lymph node metastasis				0.821			0.918			0.839
No	40	24	60.0%		13	32.5%		14	35.0%	
Yes	54	33	61.1%		16	29.6%		20	37.0%	
P-TNM stage				0.632			0.628			0.719
Ⅰ+Ⅱ	53	31	58.5%		18	34.0%		20	37.7%	
Ⅲ	41	26	63.4%		11	26.8%		14	34.1%	
Smoking history				0.140			0.945			0.227
No	34	24	70.6%		10	29.4%		15	32.4%	
Yes	60	33	55.0%		19	31.7%		19	31.7%	

### 2.3 EGFR阳性患者中c-Cbl、Cbl-b的表达与临床病理参数之间的关系

94例NSCLC组织标本中有57例患者存在不同程度的EGFR蛋白表达，亚组分析发现此57例患者中，c-Cbl、Cbl-b的表达与性别、年龄、病理类型及TNM分期等参数无关（表 2）。

**2:** 在EGFR阳性的患者中c-Cbl、Cbl-b的表达与临床病理特征的关系 Relationship between c-Cbl and Cbl-b expression and clinical pathophysicological characteristic of EGFR positive NSCLC

Parameter	*n*	c-Cbl		*P*	Cbl-b		*P*
		Positive	Rate		High expression (%)	Rate	
Age (year)				0.278			0.832
< 60	37	14	37.8%		14	37.8%	
≥60	20	4	20.0%		7	20.0%	
Sex				0.819			0.658
Male	40	13	32.5%		14	35.0%	
Female	17	5	29.8%		7	41.2%	
Histology				0.518			0.812
Squamous cell carcinoma	25	9	36.0%		9	36.0%	
Adenocarcinoma	28	8	28.6%		10	35.7%	
Large cell carcinoma	3	1	33.3%		2	66.7%	
Adenosquamous cell carcinomas	1	0	0.0%		0	0.0%	
Differentiated				0.999			0.999
Highly &modrately	38	12	31.6%		14	36.8%	
Poorly	19	6	33.3%		7	33.3%	
Tumor size				0.245			0.448
T1+T2	40	15	37.5%		16	40.0%	
T3+T4	17	3	17.6%		5	29.4%	
Lymph node metastasis				0.412			0.640
No	24	9	27.3%		8	33.3%	
Yes	33	9	37.5%		13	39.4%	
P-TNM stage				0.066			0.384
Ⅰ+Ⅱ	31	13	41.9%		13	41.9%	
Ⅲ	26	5	19.2%		8	30.8%	
Smoking history				0.738			0.930
No	24	7	29.2%		9	37.5%	
Yes	33	11	33.3%		12	36.4%	

### 2.4 EGFR、c-Cbl、Cbl-b与预后的关系

生存分析显示EGFR、c-Cbl及Cbl-b的表达水平对患者的预后无影响（图 2）。亚组分析（图 3）发现EGFR阳性患者中Cbl-b高表达组总生存有优于Cbl-b低表达组患者的趋势，但无统计学差异（*P*=0.138）；c-Cbl阴性组患者的中位生存时间为（38.1±4.1）个月，c-Cbl阳性组患者的中位生存时间为（59.7±5.8）个月，两组差异有统计学意义（*P*=0.014）。*COX*多因素分析发现肿瘤大小、淋巴结转移是影响预后的独立危险因素。

**2:**
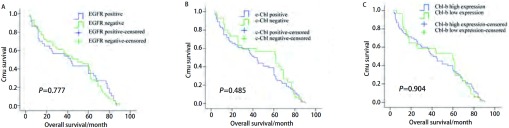
EGFR、c-Cbl、Cbl-b的表达与患者生存之间的关系。A：EGFR；B：c-Cbl；C：Cbl-b。 Survival curves of NSCLC patients with expressions of EGFR, c-Cbl and Cbl-b. A: EGFR expression (*P*=0.777); B: c-Cbl expression (*P*=0.485); C: Cbl-b expression (*P*=0.904).

**3:**
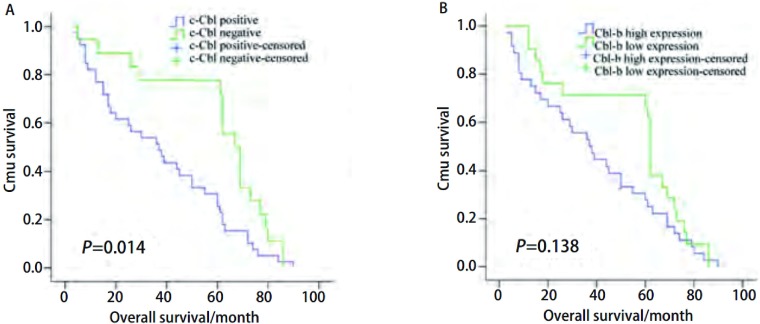
EGFR阳性组c-Cbl、Cbl-b的表达与患者生存之间的关系。A：c-Cbl；B：Cbl-b。 Survival curves of EGFR positive NSCLC patients classified by C-Cbl and Cbl-b. A: c-Cbl expression (*P*=0.014); B: Cbl-b expression (*P*=0.138).

## 3 讨论

NSCLC发生率占肺癌总发生率的80%。虽然肺癌的化疗、放疗等治疗方法有所改进，但肺癌的5年生存率并没有得到明显改善^[9]^。由于EGFR在肿瘤细胞的生长、修复和存活等方面具有极其重要的作用，它的过度表达通常预示患者预后差、转移快、对化疗耐药、生存期较短等^[10, 11]^。以EGFR为靶点的分子靶向治疗已经广泛应用于临床^[12-14]^。既往研究^[15]^发现肺腺癌中EGFR的阳性率为65%, 大细胞肺癌中EGFR的阳性率为68%。本研究结果显示NSCLC中EGFR表达的阳性率为60.64%，与既往的报道相近。女性患者阳性率高于男性，并且EGFR表达与年龄、病理类型、TNM分期及淋巴结有无转移、吸烟史等无关，可能与肺癌在人群中的性别分布有关，也可能与入组的性别偏倚有关（94例患者中男性占73例，女性占21例）。

针对EGFR的靶向治疗的疗效与EGFR的突变状态有关而与EGFR表达的高低无关，EGFR表达水平的高低对NSCLC患者化疗敏感性及预后的预测价值目前尚存在争议^[15, 16]^。近年的研究^[17, 18]^显示联合检测EGFR和其它的相关蛋白可能预测肺癌治疗的疗效及患者的预后。c-Cbl及Cbl-b是Cbl家族中的两个重要成员，能靶向调节包括以EGFR为代表的一系列具有酪氨酸激酶活性的受体蛋白使之降解，参与细胞内信号转导的负向调控，在维持机体稳态中发挥重要作用。最新研究^[19]^发现血液系统肿瘤中Cbl家族的突变导致酪氨酸激酶的增殖信号未能及时下调，进而导致患者预后不良。研究^[7]^发现c-Cbl、Cbl-b在NSCLC中存在高突变率，并推测该家族可能参与了NSCLC的发生与转移，但Cbl家族在NSCLC中表达的高低尚未见报道。本研究首次检测其在NSCLC中的表达，发现其表达率分别为30.85%和84.04%，c-Cbl蛋白表达与肿瘤大小（T分期）虽无明显关系，但存在负相关趋势（*r*=-0.192, *P*=0.078），提示患者体内c-Cbl表达水平可能会影响肿瘤的增殖。

既往实验研究^[8]^发现NSCLC中Cbl家族可通过介导EGFR的降解而终止细胞的增殖信号，但尚无相关的临床报道。本研究发现在总人群中Cbl家族的表达与患者的预后无关，但在EGFR阳性组中c-Cbl阳性的患者总生存明显优于c-Cbl阴性的患者，该结果为既往的基础研究提供了临床证据。本研究未发现c-Cbl蛋白与EGFR蛋白表达水平之间的相关性，可能因为患者数量有限，以及免疫组化本身是一种定性、非定量检测，敏感度有限，此外也有可能因为Cbl主要是通过下调EGFR的活化抑制细胞增殖。本研究发现c-Cbl和Cbl-b的表达呈正相关，提示两者之间可能存在相互的调节作用。

EGFR在肺癌的发生及发展过程中发挥着重要的功能，目前针对EGFR的靶向治疗在肺癌的治疗中取得了较好的疗效，Cbl家族作为下调EGFR的重要分子是否会成为肺癌治疗中一个新的靶点以及预测EGFR阳性肺癌患者预后的指标还有待于进一步的研究。

## References

[1] Erman M, Grunenwald D, Penault-Llorca F (2005). Epidermal growth factor receptor, HER-2/neu and related pathways in lung adenocarcinomas with bronchioloalveolar features. Lung Cancer.

[2] Franklin WA, Veve R, Hirsch FR (2002). Epidermal growth factor receptor family in lung cancer and premalignancy. Semin Oncol.

[3] Ryan PE, Sivadasan-Nair N, Nau MM (2010). The N terminus of Cbl-c regulates ubiquitin ligase activity by modulating affinity for the ubiquitinconjugating enzyme. J Biol Chem.

[4] Huang F, Gu H (2008). Negative regulation of lymphocyte development and function by the Cbl family of proteins. Immunol Rev.

[5] Tan YH, Krishnaswamy S, Nandi S (2010). CBL is frequently altered in lung cancers: its relationship to mutations in MET and EGFR tyrosine kinases. PLoS One.

[6] Duan L, Raja SM, Chen G (2011). Negative regulation of EGFR-Vav2 signaling axis by Cbl ubiquitin ligase controls EGF receptor-mediated epithelial cell adherens junction dynamics and cell migration. J Biol Chem.

[7] Sorkin A, Goh LK (2008). Endocytosis and intracellular trafficking of ErbBs. Exp Cell Res.

[8] Shtiegman K, Kochupurakkal BS, Zwang Y (2007). Defective ubiquitinylation of *EGFR* mutants of lung cancer confers prolonged signaling. Oncogene.

[9] Parkin DM, Bray Fl, Devesa SS (2001). Cancer burden in the year 2000. The global picture. Eur J Cancer.

[10] Steins MB, Reinmuth N, Bischoff H (2010). Targeting the epidermal growth factor receptor in non-small cell lung cancer. Onkologie.

[11] Wykosky J, Fenton T, Furnari F (2011). Therapeutic targeting of epidermal growth factor receptor in human cancer: successes and limitations. Chin J Cancer.

[12] Press MF, Lenz HJ (2007). EGFR, HER2 and VEGF pathways: validated targets for cancer treatment. Drugs.

[13] Vivanco I, Mellinghoff IK (2010). Epidermal growth factor receptor inhibitors in oncology. Curr Opin Oncol.

[14] Mok TS, Wu YL, Thongprasert S (2009). Gefitinib or Carboplatin–Paclitaxel in pulmonary adenocarcinoma. N Engl J Med.

[15] Hirsch FR, Scagliotti GV, Langer CJ (2003). Epidermal growth factor family of receptors in preneoplasia and lung cancer: perspectives for targeted therapies. Lung Cancer.

[16] Nicholson RI, Gee JM, Harper ME (2001). EGFR and cancer prognosis. Eur J Cancer.

[17] Mukohara T, Kudoh S, Yamauchi S (2003). Expression of epidermal growth factor receptor (EGFR) and downstream-activated peptides in surgically excised non-small-cell lung cancer (NSCLC). Lung Cancer.

[18] Argiris A, Hensing T, Yeldandi A (2006). Combined analysis of molecular and clinical predictors of gefitinib activity in advanced non-small cell lung cancer: epidermal growth factor receptor mutations do not tell the whole story. J Thorac Oncol.

[19] Makishima H, Cazzolli H, Szpurka H (2009). Mutations of e3 ubiquitin ligase cbl family members constitute a novel common pathogenic lesion in myeloid malignancies. J Clin Oncol.

